# Design of a combinatorial DNA microarray for protein-DNA interaction studies

**DOI:** 10.1186/1471-2105-7-429

**Published:** 2006-10-03

**Authors:** Julian Mintseris, Michael B Eisen

**Affiliations:** 1 Boston University, Bioinformatics Program, Boston, MA, USA; 2 Department of Genome Sciences, Lawrence Berkeley National Lab, Berkeley, CA, USA; 3 Department of Molecular and Cell Biology, University of California, Berkeley, CA, USA

## Abstract

**Background:**

Discovery of precise specificity of transcription factors is an important step on the way to understanding the complex mechanisms of gene regulation in eukaryotes. Recently, double-stranded protein-binding microarrays were developed as a potentially scalable approach to tackle transcription factor binding site identification.

**Results:**

Here we present an algorithmic approach to experimental design of a microarray that allows for testing full specificity of a transcription factor binding to all possible DNA binding sites of a given length, with optimally efficient use of the array. This design is universal, works for any factor that binds a sequence motif and is not species-specific. Furthermore, simulation results show that data produced with the designed arrays is easier to analyze and would result in more precise identification of binding sites.

**Conclusion:**

In this study, we present a design of a double stranded DNA microarray for protein-DNA interaction studies and show that our algorithm allows optimally efficient use of the arrays for this purpose. We believe such a design will prove useful for transcription factor binding site identification and other biological problems.

## Background

With the human and many other genome sequences complete or nearing completion, we are approaching the goal of identifying all the protein coding genes. However, to understand the function of these genes in different physiological contexts, it is important to understand how their expression is regulated. Mechanisms of gene regulation are varied and complex and unraveling them will require a combination of approaches[1,2]. Having a catalog of all the transcription factors and being able to characterize their binding specificity at *cis*-regulatory sites would provide a fruitful starting point.

Recent advances in chromatin immunoprecipitation (CHIP) methods have led to large-scale efforts to determine all protein-DNA binding events in yeast[3,4] but scaling up such methods for mammalian genomes may prove difficult. Protein-binding microarrays (PBM), initially developed on a small scale by Bulyk et al[5,6] showed promise in identifying transcription factor binding specificity with high accuracy and was recently successfully scaled up for the yeast genome by using PBMs with all known yeast intergenic regions[7]. Although an exciting advance in the field, current design of PBMs still leaves room for uncertainty because some of the intergenic regions may be too long to pinpoint the binding sites with high accuracy. Scaling this method up to mammalian genomes would also require designs spanning multiple arrays, with a new design for each genome. Both CHIP and PBM methods are well suited for low resolution identification of genes affected by a given transcription factor. However, in order to fully understand regulation, researchers will always be interested in pinpointing the specific regions to which the factor binds. Identifying this region from CHIP-CHIP or PBM data requires sophisticated computational analysis, much like that used in ab-initio cis-regulatory region discovery. Reliability of such analyses is sometimes questionable, in part because of the repetitive and degenerate nature of the intergenic sequences. Harbison et al. note that some intergenic sequences are highly homologous thus skewing the results of motif discovery algorithms[4]. If there was a way to test the binding of a given factor to all possible motifs of a given length, it would then be trivial to scan the intergenic sequences for potential sequences corresponding to a well-defined motif. We therefore propose a new PBM design that would allow the testing of all possible binding sequences of a given length in an optimally-efficient non-degenerate manner.

In recent years, a number of technological innovations took place, allowing programmable synthesis of microarrays as well as new techniques to make the arrays double-stranded[8,9]. In particular, Warren et al. successfully constructed and tested a combinatorial dsDNA array with all possible 8-mer sequences, with one sequence per spot[9]. Since the proof of principle for this technology has now been shown, here we focus on optimizing experimental design. Using variations on established graph theory algorithms, we propose a new design of a PBM, which would allow the *in-vitro *testing of transcription factor binding to all possible DNA targets up to length 12. This approach removes some of the redundancy in testing long intergenic regions. In addition, our design is organism-independent.

## Results

### Algorithm

The design, as described by Bulyk et al. in proof-of-concept papers [5,6] allows for testing *N *binding sites by screening *N *spots on the array. This approach is straightforward but not very practical for most transcription factors because the number of possible binding sequences is 4^*k*^, where *k *is the length of the binding site.

The more recent design involved spotting all annotated yeast intergenic regions on the array[7]. This comprehensive approach is more scaleable, although mammalian genomes contain long "desert" regions[10] which would most likely have to be broken up into shorter segments for spotting on microarrays. In order to identify the transcription factor binding sites within the spotted regions, in this as well as in many other approaches, the authors rely on a variant of the Gibbs sampling algorithm. Some of the longer intergenic regions tested may present a problem in identifying binding patterns for low-specificity transcription factors. Uniform probe length and optimal non-redundancy of the array proposed here would make it easier to analyze experimental results and estimate their statistical significance.

We propose the design of a dsDNA array that allows screening for length *k *TF binding sites with maximum efficiency by allowing the *k*-mers to overlap. For instance, the 8-mer probe ACTGTGCA represents two potential 7-mer TF binding sites – ACTGTGC and CTGTGCA. It turns out that we can easily design an array with probes of certain length b that contain all possible *k*-mers, such that the required number of probes is minimal. If we can find the shortest string that contains all possible *k*-mer substrings, we can then "cut up" this string into individual probes of desired length. The problem of constructing such a minimum-length string can be represented in graph-theoretical formulation (see Methods for details).

Imagine a directed graph with nodes represented by all possible *k*-mers, where the edges exist between nodes that overlap by (*k*-1). Finding the shortest path for a graph of all possible *k*-mers results in a superstring of length (4^*k *^+ *k*). Given a desired probe length *b *> *k*, we can design an array with *N *probes that enables us to test the binding specificity of any transcription factor that can bind to a *k*-mer. The number of probes would have to be approximately

*N *= 4^*k*^/(*b*-*k*+1)

The length of a string produced by naively joining all possible k-mers is k*4^*k*^. This means we are able to reduce the number of probes by a factor of *k*. Furthermore, we can turn the reverse complementarity of double-stranded DNA sequence to our advantage and gain another factor of 2 reduction in number of array probes[9,11]. For instance, having included the 7-mer ACTGTGC in the superstring and assuming that the array probe will be double stranded, we are already accounting for the reverse complement 7-mer GCACAGT. This introduces some complications in the algorithm, which we discuss in Methods.

**Figure 1:**
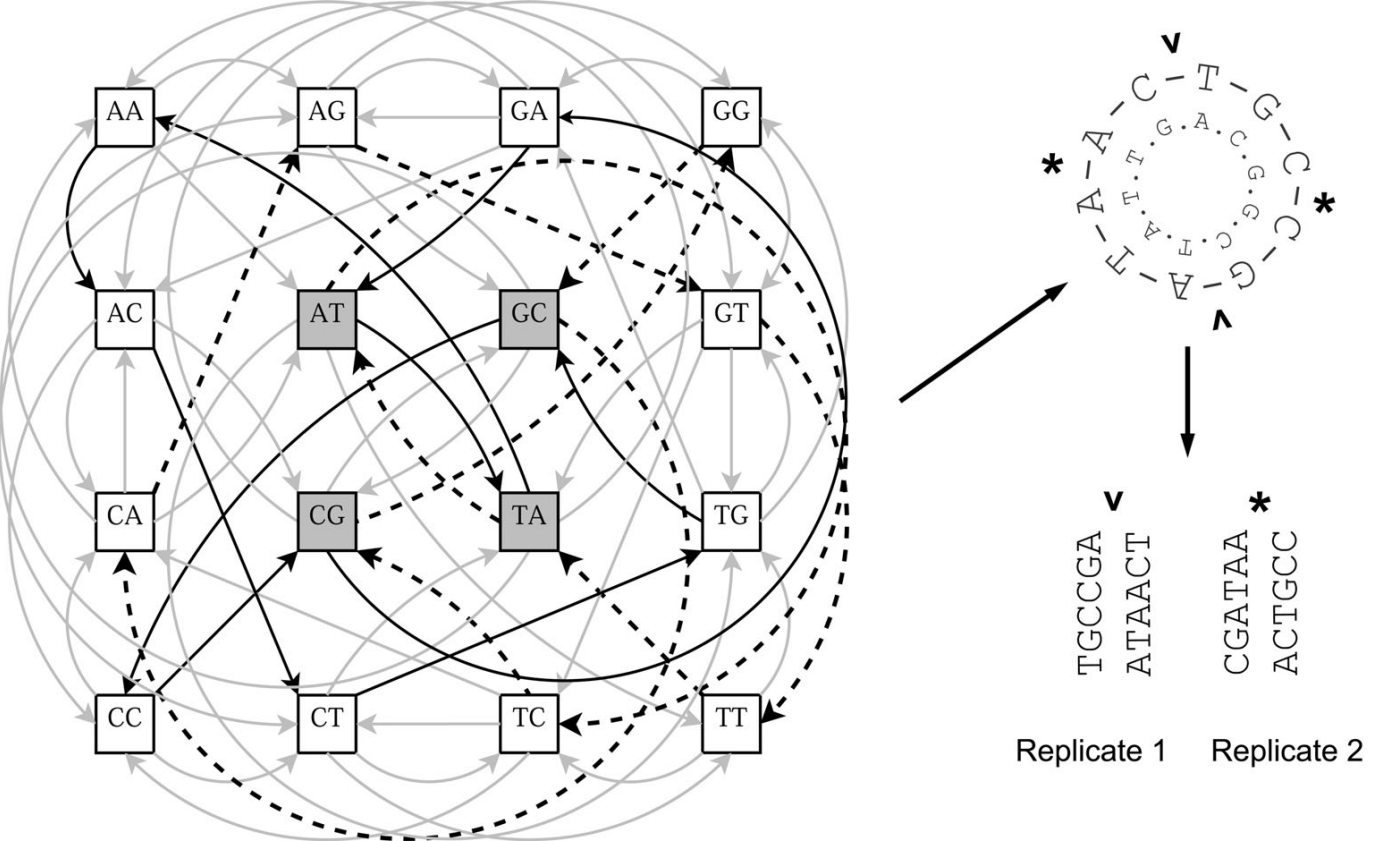
**Probe design from the shortest path on a graph**. The de Bruijn graph for all possible DNA base doublets and one possible solution for a shortest path represented as a pseudo-Eulerian cycle (bold edges). The reverse complement solution is represented by dashed edges in the graph and also the inner cycle sequence. "Cutting" the circular sequence while retaining one overlapping base results in two sequences of total length 12 (containing all doublets) as compared to the length of all non-overlapping concatenated doublets 2 * 4^2 ^= 32. Cutting the circular sequence at different points allows screening multiple replicates and helps identify biases in sequence recognition preferences. Reverse complement strands for the replicates are not shown.

Figure 1 shows the graph and the resulting "probes" for the simplest case, where *k *= 2. Here, we save approximately a factor of 4 of the length of DNA to be tested, but for all possible 10-mers, we would save a factor of ~20.

We would also need to take into account some additional considerations, such as allowing for spacers on either side of the designed sequence to ensure reliable binding, as well as a primer, if the double stranded DNA is constructed enzymatically. We believe such an approach takes some of the ambiguity out of the decoding process that is needed in current approaches that rely on spotting long intergenic regions[7].

### Experimental design

Using our combinatorial design, testing of all possible 10-mers with an array of probes of length 25 (not including any spacers or primers) requires only 32928 probes. To avoid potential problems with factors binding to multiple sites on a given probe, and to aid in the identification of precise binding sites, the experiment may be performed in duplicate, with the cut points on the cyclical superstring shifted by k/2 (Figure 1). Table 1 shows the calculations for the number of probes needed on the array for a range of motif lengths k and array probe lengths b.

**Table 1:** Sample calculations for the number of probes/array

**Probe Length b**	**Motif Length k**							
	**5**	**6**	**7**	**8**	**9**	**10**	**11**	**12**
**25**	25	108	432	1849	7711	32928	139811	600064
**30**	20	86	342	1447	5958	25088	104858	442153
**35**	17	72	283	1189	4855	20264	83887	350038
**40**	15	62	241	1009	4096	16996	69906	289687
**45**	13	54	211	876	3543	14635	59919	247086
**50**	12	48	187	774	3121	12850	52429	215408
**55**	11	43	168	694	2789	11454	46604	190930
**60**	10	39	152	628	2521	10331	41944	171447
**65**	9	36	139	574	2300	9408	38131	155573
**70**	8	33	128	529	2115	8637	34953	142389

Identifying the actual binding sequences given intergenic array spot data is a non-trivial problem, which Mukherjee et al. addressed by Gibbs-sampling algorithms[7,12]. This problem arises from a combination of two factors: 1) many intergenic sequences are quite long (mean length 486 bp for yeast), increasing the probability of finding multiple binding sites; 2) intergenic sequences are inherently redundant. Our combinatorial design addresses both of these issues by proposing reasonably short and optimally non-redundant sequence features.

**Figure 2:**
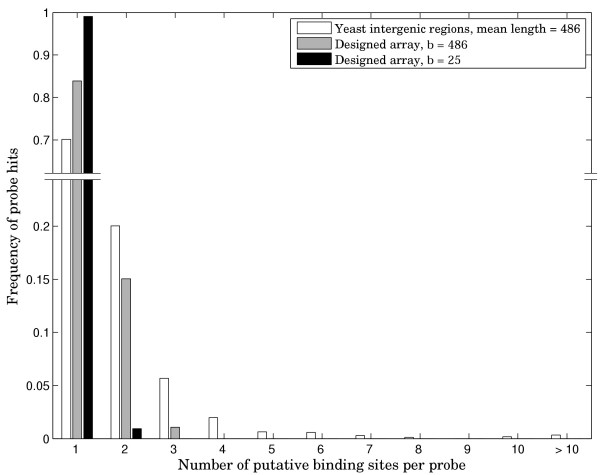
**Distribution of putative PBM probe hits for Rap1**. Frequency of array probe hits distributed by number of potential binding sites per probe. All sequences one or two mutations away from the consensus sequence are assumed to bind.

**Figure 3:**
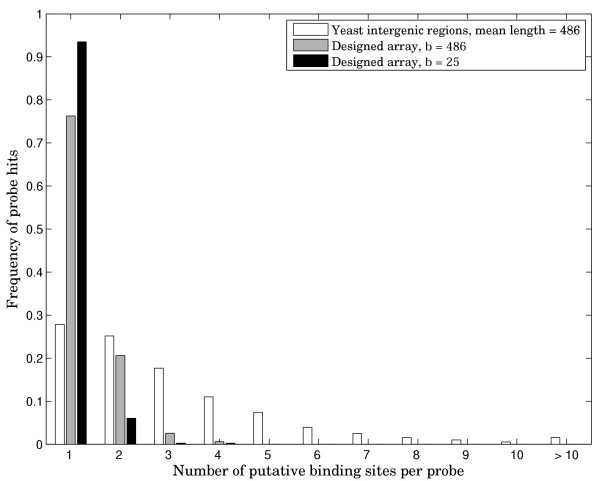
**Distribution of putative PBM probe hits for TBP**. Frequency of array probe hits distributed by number of potential binding sites per probe. All sequences one or two mutations away from the consensus sequence are assumed to bind.

**Figure 4:**
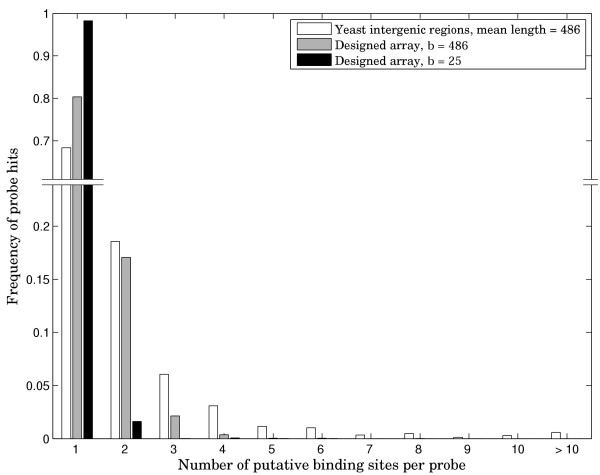
**Distribution of putative PBM probe hits for 100 random transcription factor binding sites of length 10**. Frequency of array probe hits distributed by number of potential binding sites per probe. The data is averaged over 100 random 10-mer binding sites. For each 10-mer, all sequences one or two mutations away from the consensus sequence are assumed to bind.

In order to illustrate the advantage of our design in more precisely identifying the exact binding sequences, we carried out simulation experiments with yeast Rap1 transcription factor, yeast TATA-Box Binding Protein (TBP), as well as 100 random binding sites of length 10. Since some transcription factors are known to tolerate substantial variation of the binding site sequence, we generated all possible double mutants for every starting consensus binding site sequence and assumed that all those sequences would be recognized on the array. For our designed array, we chose a design from Table 1 with k = 10 and b = 25. Because a probe of length 25 is statistically much less likely to contain multiple binding sites for a given factor than a probe of length 486, we also included a combinatorial design with b = 486. Note that synthesis of a dsDNA array with feature length of 486 would be very difficult if not impossible and is only used here to illustrate the properties of combinatorial design. The results of these simulations are presented in Figures 2, 3, 4. The simulation data shows that for Rap1 and for random 10-mers, about 20–30% of intergenic PBM probes producing signal on the array in fact contain more than one binding site. This figure is greater than 70% for the more degenerate TATA-box sequence. In all cases, the designed array, even with average probe length of 486 results in significantly fewer multiple site probes, showing that non-redundancy comes from our combinatorial design and not just from the reduced probe length. Furthermore, results for the designed array with 25-mer probes are good enough that in doing the array analysis, one can assume a single binding event per probe. Rap1 and the averaged data for 100 random sequences show ~1–2% multiple binding sites per probe. The TBP simulation results in ~6.5% putative multiple binding events.

### Signal-to-noise ratio

**Figure 5:**
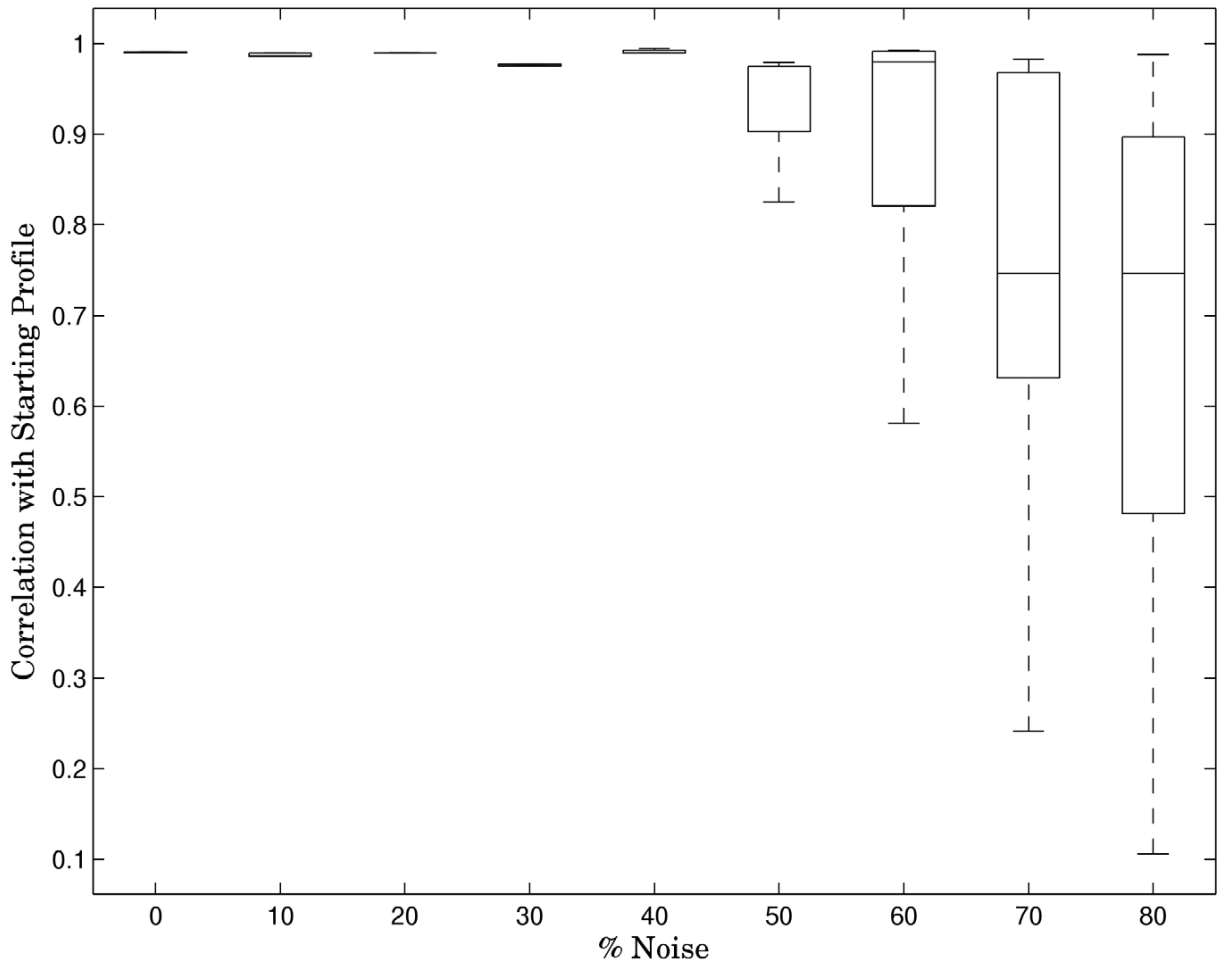
**Robustness of designed array and Gibbs Sampler to addition of noise**. Starting with a set of 10-mer Rap1 TRANSFAC binding sites, the effect of added noise is measured as correlation of the original PWM with that derived from 100 Gibbs Sampler-runs. Each level of noise is represented by the standard box-and-whisker plot. In the 0–50% noise range, the boxes are so small that they are essentially represented by a single line.

As mentioned above, the problem of finding precise binding sites in long intergenic sequences used in CHIP and PBM experiments, is traditionally addressed by Gibbs-sampling and related algorithms. The reasons why Gibbs sampling algorithms do not always perform well fundamentally come down the ratio of signal to noise in the dataset in question. This ratio can be estimated as the number of base-pairs involved in binding divided by the total number of base-pairs in the array probe. Since the number of binding site bases in the combinatorial design remains approximately the same, and the total probe length decreases from a mean of 486 bp to 25, we can estimate that our design reduces the signal-to-noise ratio by at least an order of magnitude. Indeed, finding a 10-mer binding site in a set of 25-mers is almost a trivial Gibbs sampling problem. In order to test the robustness of our designed array to experimental noise, we constructed a 10 bp wide PWM (Position Weight Matrix) of the Rap1 transcription factor from TRANSFAC[13] data, containing 14 distinct aligned sequences. Assuming, for testing purposes, that these sequences represent the entire set of Rap1 targets, we found all the combinatorial array probes and those of one replicate (see Figure 1 and legend) that included those sequences. We then proceeded to remove a fraction of these sequences from the probe set and substitute for them random probes, not containing the binding site. Upon each iteration, we used BioProspector[14], a popular implementation of the Gibbs sampling algorithm, to scan the sequences 100 times and find an overrepresented motif. We then used CompareACE[15] to calculate the correlation coefficient between the obtained motif and the original PWM that we started with. The results are presented in Figure 5. The motif extracted with the Gibbs sampler remains essentially identical to the original, withstanding up to 50% substituted noise.

### Flanking sequences

The early versions of PBMs were made double-stranded by enzymatic primer extension, [5,8] which would mean that the combinatorial portion of the probe intended to assay for protein binding would be adjacent (either 3' or 5') to a constant primer sequence. Of course, any such primer sequence could also contain a portion of a binding site or even an entire binding site, making it difficult to analyze the data. The more recent approach involved only a short 3-base flanking sequence on either side of the combinatorial portion of the probe, thus eliminating the problem[9]. Nevertheless, the enzymatic primer extension approach remains a valid option and has the advantage of higher fidelity, compared with oligo synthesis. It is therefore important to address the potential challenge of analyzing data from an experiment where the flanking sequence is bound on some probes and deciphering the true binding site in such an experiment.

We propose that this challenge be addressed by making a replicate array (Figure 1). The simplest approach would be to make a replicate array with different primers/flanking sequences. If the number of bound probes differs significantly between the two replicates, it would suggest that the flanking sequence is involved in one of them. Analysis of the array with the smaller number of bound probes should reveal the true binding site and help extract additional information from the other replicate.

Even with constant flanking sequence, we could solve the problem by making one or more non-identical array replicates obtained by "shifting" the probe cut sites on the superstring sequence as illustrated in Figure 1. The advantage of such replicate design is that, while the set of k-mers on the array remains the same, the position of each k-mer with respect to the chip surface is different. Table 2 contains simulated examples for the case when half of the Rap1 consensus binding site (CACCCATACA) is contained in the flanking primer sequence of the probe, thus allowing for a large number of possibilities matching in the combinatorial part of the probe. We can filter the matching probes, retaining only those replicate probe pairs that contain at least one combinatorial k-mer in common with each other. If the flanking sequence contained a portion of the binding site, the number of probes should decrease substantially after filtering, otherwise most of the probes will be retained (Table 2). For cases when a portion of the flanking sequence is involved in binding, the filtering procedure will also retain some randomly paired probes but because the signal-to-noise ratio is high, the true binding site can still be easily detected by Gibbs sampling.

**Table 2:** Using array replicates to discover the Rap1 binding site when the flanking sequence is involved in binding.

**Flanking/Primer Sequence^a^**	||xxxxxxxxxxx xxxxxxxxxxxxxxxx primer combinatorial	||xxxxxx**CACCC **xxxxxxxxxxxxxxxx primer combinatorial
**Total # of Probes Bound**	29	744
**Top BioProspector Hits (1^st ^n)**	CACCCATACA (34)	ATTCATGCTC (1)
**# of Replicate Probes Bound**	28	59
**Top BioProspector Hits (1^st ^n)**	CACCCATACA (37)	CACCCATACA (25)

^a ^The first array design contains a flanking primer sequence that does not contain any part of the binding site. In the second array design, the last 5 bases of the flanking primer sequence (shown in bold) constitute half of the consensus Rap1 binding site.

## Discussion

While the technological aspects of array construction have been the subject of much recent work, less attention has been paid to the oligonucleotides on these arrays in terms of experimental design. Here we have laid out an algorithmic solution to the design of a DNA microarray that would allow the characterization of binding specificity of any transcription factor independent of the species under study. The solution discussed here focuses on the algorithmic part of the problem and does not include some of the concerns involved in the production of such an array. However, we believe that given the recent advances in microarray technology, the arrays described here are well within the reach of current state of the art. Custom arrays can be obtained from several sources such as Agilent, Nimblegen[16] and several others and new technologies for programmable array synthesis are still being developed[17]. Synthesis of the complementary strand on the arrays can be achieved enzymatically with a surface-proximal primer[5] or with other, more recently developed methods[8,9].

Analysis of intergenic PBM data has been complicated by the fact that the sequences are long, redundant, and often contain multiple binding sites especially for factors that do not bind with high specificity. Our design addresses this problem and in simulations produces data that is much easier to analyze due to higher signal-to-noise ratio. Given our simulation data, it seems reasonable to make the assumption of a single binding site per probe and thus make it much easier for Gibbs sampling algorithms to converge on the correct solution.

The combinatorial array design that includes all possible k-mers also has the advantage that as genome annotation continues to improve, including the validation of intron/exon boundaries and discovery of novel genes, the data obtained from such an array remains valid and relevant.

Despite the probe number savings offered by the design presented here, the exponential growth of the number of probes as a function of k will limit the length of combinatorial binding sites. However, even with k up to 12, the design can be applied to many important unresolved problems. Applications of ideas presented here extend beyond transcription factor interactions. For instance, they may also prove useful to characterize restriction enzyme specificity, DNA methylation patterns and in other systematic studies. The array could be used to study not only the binding patterns of natural DNA-binding proteins, but also to analyze mutants and thus help us gain a more detailed understanding of the nature of specificity/promiscuity of these interactions as well as design new ones.

## Conclusion

In this study, we present the design of a microarray containing all combinations of a DNA motif for testing of transcription factor binding and other protein-DNA interaction applications. The advantage of this approach is that it is exhaustive and the same exact design could be used for any genome. Furthermore, uniform probe lengths and optimal non-redundancy allows for a more straightforward statistical analysis of the results. Combined with recent advances in PBM technology development,[9] our design will enable more precise identification of true binding sites.

## Methods

The problem of constructing a minimum-length string can be represented in graph-theoretical formulation. Imagine a directed graph with nodes represented by all possible *k*-mers, where the edges

<u,v> exist iff *u *= *s*_1_*s*_2 _... *s*_*n*-1 _and *v *= *s*_2 _... *s*_*n*-1_*s*_*n*_

Then, walking the shortest path through this graph results in the construction of the shortest cyclical sequence that contains all the subsequences only once. This turns out to be a well-known problem in computer science known as the Chinese Postman problem. The shortest path visiting the edges only once is known as the Eulerian cycle. Moreover, the problem is specifically known in terms of constructing the minimal string sequence known as the de Bruijn sequence. The graph consisting of all possible subsequences of a certain length from an alphabet of a given size is known as the de Bruijn graph. A Eulerian path is easily found in linear time with Fleury's algorithm[18].

The algorithm has to be modified to take advantage of the fact that for a double-stranded DNA probe, every k-mer in the probe will also have a reverse complement and therefore, the reverse complement sequence optimally should not be included in the superstring. Every de Bruijn graph therefore contains within it two "reverse complementary" sub-graphs. There is an additional complication arising from the fact that graphs with *k *= even and *k *= odd have different properties. Constructing the minimal superstring for odd-*k *graphs amounts to finding two "pseudo-Eulerian" cycles, which are reverse complementary to each other. This can be achieved simultaneously in the context of Fleury's algorithm. Even-*k *graphs are further complicated by the fact that some nodes are reverse complements of each other (e.g. ACGT) and are therefore shared nodes between the two reverse complementary sub-graphs. Because of this peculiarity, the number of nodes in a "pseudo-Eulerian" cycle containing each k-mer or its reverse complement only once is equal to *k*/2 for odd *k *graphs and slightly more than *k*/2 for even *k *graphs. As shown in Figure 1, this comes from the fact that k-mers that are reverse complements of each other have to be counted twice – once for each of the reverse-complementary sub-graphs. The figure shows two possible "pseudo-Eulerian" reverse-complementary cycles for *k *= 2, with the four self-complementary nodes highlighted.

In simulation to test how robust the array probes are to noise, BioProspector software was run to try to find a motif 100 times per run, using the probe sequences from the entire designed array as background.

In primer/flanking sequence simulations, we used ACTGACGTACTGGTTT as a control primer (not containing a part of Rap1 binding site) and ACTGACGTACT**CACCC **as the primer sequence with the last 5 bases overlapping the Rap1 consensus binding site (CACCCATACA).

## Authors' contributions

JM and MBE conceived and designed the study. JM carried out the study and drafted the manuscript. All authors read and approved the final manuscript.
